# Analysis of Gamma-Band Activity from Human EEG Using Empirical Mode Decomposition

**DOI:** 10.3390/s17050989

**Published:** 2017-04-29

**Authors:** Carlos Amo, Luis de Santiago, Rafael Barea, Almudena López-Dorado, Luciano Boquete

**Affiliations:** Departamento de Electrónica, Grupo de Ingeniería Biomédica, Universidad de Alcalá, Alcalá de Henares 28801, Spain; carlos.amo@edu.uah.es (C.A.); luis.desantiago@uah.es (L.d.S.); rafael.barea@uah.es (R.B.); almudena.lopez@uah.es (A.L.-D.)

**Keywords:** electroencephalography, gamma-band activity, motor area, motor tasks, empirical mode decomposition, event-related synchronization, power spectral density

## Abstract

The purpose of this paper is to determine whether gamma-band activity detection is improved when a filter, based on empirical mode decomposition (EMD), is added to the pre-processing block of single-channel electroencephalography (EEG) signals. EMD decomposes the original signal into a finite number of intrinsic mode functions (IMFs). EEGs from 25 control subjects were registered in basal and motor activity (hand movements) using only one EEG channel. Over the basic signal, IMF signals are computed. Gamma-band activity is computed using power spectrum density in the 30–60 Hz range. Event-related synchronization (ERS) was defined as the ratio of motor and basal activity. To evaluate the performance of the new EMD based method, ERS was computed from the basic and IMF signals. The ERS obtained using IMFs improves, from 31.00% to 73.86%, on the original ERS for the right hand, and from 22.17% to 47.69% for the left hand. As EEG processing is improved, the clinical applications of gamma-band activity will expand.

## 1. Introduction

### 1.1. Electroencephalogram (EEG)

An electroencephalogram (EEG) represents the electrical activity of the brain, recorded by placing several electrodes on the scalp. This activity is generated by the dendrites of the neurons adjacent to the cortical surface. The EEG frequency range is classified into neural oscillatory patterns: delta (1–4 Hz), theta (4–8 Hz), alpha (8–12 Hz), beta (15–30 Hz), and gamma-band oscillations (>30 Hz) [1].

One of the acknowledged issues with EEG is its low amplitude (on the order of microvolts) and high noise. Muscular artifacts, eye movements, and cardiac pulse are the principal sources of contamination in EEG recordings [2]. Semiautomatic detection of artifacts (eye blinks, muscle contraction, etc.) methods improve the quality of analysis results of EEG signals [3,4,5]. Signal processing methods, such as extraction of power spectral density (PSD) [6], wavelet analysis [7], independent component analysis (ICA) [8], and local mean decomposition [9], are used to improve recording quality and to analyze data. In most cases, automatic classification methods are implemented [10,11,12,13].

EEG is used extensively in neuroscience, cognitive science, cognitive psychology, neurolinguistics, and psychophysiological research. This is in addition to its more traditional place in clinical assessments or consciousness research [8].

The typical frequency range employed for the diagnosis of various brain conditions are low frequency bands (delta, theta, and alpha). Recently, new horizons have been explored by extracting and analyzing high frequency (>30 Hz) components of EEG signals, applied to clinical assessments in diseases such as schizophrenia, visual processing deficits, and motor cortex dysfunctions. Robust results have been obtained using PSD computing in the frequency (>30 Hz) band [6,14,15].

Multichannel EEG systems are commonly employed to obtain high spatial resolutions. However, the setup process is time-consuming (attaching the electrodes to the scalp, adjusting the skin–electrode impedance value by adding gel). In addition, the system is uncomfortable and limits the movements of subjects. In ambulatory [2] or in portable-wearable applications, single-channel EEG systems are the best choice as they preserve brain activity very well, while also being easy to use. In recent years, acquisition and measurement techniques have been increasingly deployed in monochannel systems: dry electrodes, hardware systems, or new processing algorithms [16,17,18]. EEG monochannel systems are broadly used in several applications, such as brain computer interfaces [19], sleep studies [20,21], indicators of hypoxia [22], etc.

### 1.2. Gamma Activity

Gamma-band activity (GBA) comprises an EEG frequency range, from 30 to 200 Hz, and is distributed widely throughout cerebral structures. GBA participates in various cerebral functions, such as perception, attention, memory, consciousness, synaptic plasticity, and motor control [23].

Within the gamma-band frequency range, it is possible to differentiate between low gamma-band oscillations (30–60 Hz) and high gamma-band oscillations (60–200 Hz) [24].

Some electrophysiological studies have shown that gamma-band activity in the frequency range of 30 to 90 Hz can be recorded from a wide range of brain regions during rest or the performance of motor tasks [25,26].

During sensory, cognitive, and motor processes, two types of changes may be produced via electrical activity in the cerebral cortex. The first, evoked activity, is associated with stimulus and is time and phase locked. This evoked activity can be extracted from background activity in EEGs using linear methods, such as averaging. The second change is induced activity, associated with stimulus, and is time locked but not phase locked. Induced activity can only be extracted from background activity in EEGs using non-linear methods, such as power spectral analysis [27]. These non-linear methods use time–frequency transformations (e.g., Morlet wavelets or short-time Fourier transforms) on each individual trial prior to averaging across all trials [28].

Gamma bands were reported with visual stimulation and in movement or motor tasks [8]. The typical method to quantify induced GBA is comprised of the following steps: (a) band pass filtering and artifact rejection; (b) computing the power spectral density for a selected frequency range; and (c) quantifying the variations in GBA that are induced by a motor task (motor GBA) in relation to basal cortical activity (basal GBA) [29]. The decreases or increases in motor GBA, in relation to the basal level, are known as event-related desynchronization (ERD) and event-related synchronization (ERS), respectively [30]. The interpretation of ERD and ERS in the gamma band is related to a binding of sensory information and sensorimotor integration.

Gamma band activity is typically registered with a high number of electrodes: 128 [31] for the study of neural oscillations and event-related potentials in the sound-induced flash illusion in schizophrenia; 64 [32] for the assessment of tonic muscle pain; 32 [33] for multisensory processing deficits in patients with schizophrenia; or 30 [34] for computing sensory-evoked and event-related gamma coherences in Alzheimer’s disease. This implies a long duration for the placement of electrodes and adjusting contact impedance. Complex signal processing methods must be used to reduce the noise and amplify components (band pass filters, artifact rejection, ICA, wavelets). Analyses are based on power spectral density computing in the band of interest using time-frequency analyses.

### 1.3. Empirical Mode Decomposition (EMD)

Since its presentation by Huan et al. in 1998 [8], EMD has been applied to study the non-linear and non-stationary properties of time series in areas such as geophysical studies [35], image analysis [36], thermal profiles analysis [37], and power quality analysis [38]. EMD has demonstrated itself to be a reliable and effective method in the processing of different biomedical signals, such as EEGs [9], electromyography [39], visual evoked potentials [40], de-noising of ECG signals [11], and EEG artifact removal [2].

Few works have used EMD to study neural oscillatory pattern activity associated with tasks. In Reference [41], EMD was applied as a frequency filter to separate different frequency bands. In Reference [42], EMD was computed to obtain mu and beta rhythms in assessing motor imagery movements. Theta oscillation was selected using EMD in Reference [43]. In Reference [44], the authors demonstrated a better localization of time-varying frequency components of mu and beta rhythms during motor imagery using the EMD algorithm. In their following work [45], they used an extension of the algorithm of EMD, named multivariate EMD (MEMD), to circumvent the problem of cross-channel interdependence in a 64-channel setup. In this work, the gamma rhythm, decomposed using MEMD, showed a high correlation with the eventual movement accuracy.

### 1.4. Study Objectives

The main goal of this work was to investigate whether EMD monochannel EEG signal decomposition increased the detection of gamma-band activity of motor tasks. The original EMD method was applied as only one channel was used. This eliminated cross-channel interdependencies, avoiding the uniqueness problem [45]. As EEG processing was improved, the clinical applications of GBA were extended.

Because gamma activity is associated with a cognitive task-induced brain, the subjects of the experiment were induced to generate gamma band activity.

## 2. Materials and Methods

The study protocol was approved by the Ethics Committee of the Universidad de Alcalá (Madrid, Spain). Subjects participating in the study were required to sign an informed consent form prior to the start of the experiment.

### 2.1. Sample

The sample for this experiment was comprised of 31 subjects, six of whom were excluded during data analysis due to the presence of a high number of artifacts in their EEG recordings (blinking, muscle artifacts, etc.). All sample subjects were healthy, and none of the subjects were taking drugs or had a record of alcohol or drug abuse or dependency.

The final sample was comprised of 9 females and 16 males (mean age = 25.16; range = 18–47). The subjects were classified by manual laterality, according to the Edinburgh Handedness Inventory (EHI) [46], identifying 19 right-handed subjects (mean EHI = 79.47), 3 left-handed subjects (mean EHI = −73.33), and 3 ambidextrous subjects (mean EHI = 20.00).

The recording procedures were explained in detail in a previous publication [29]. Briefly, the experiment was comprised of two parts. In the first part (basal experiment), the subjects kept their eyes open and their gaze fixed on the center of a computer screen. A total of 18 min of basal activity was recorded. The objective of this first phase was to obtain basal GBA (spontaneous gamma oscillations) from the EEG trace.

In the second phase (motor experiment), the subjects performed a simple motor task immediately after receiving an on-screen cue, thereby obtaining motor GBA, induced by that movement (induced gamma oscillations), from the EEG trace. The task consisted of rapidly bending the wrist upwards and then briefly relaxing (rather than voluntarily flexing it). This task was performed for both hands and was organized into trials of 2 s of duration. Each trial started with the display of the cue in the center of the computer screen. This was followed by a white screen that remained in place until the start of the next trial. The cue was the order to start the motor task. The motor experiment comprised five runs of 100 trials per hand. Runs alternated between the right and left hands to prevent muscle fatigue. Total motor task duration was approximately 40 min.

### 2.2. Data Acquisition

The equipment used in this experiment comprised a 32-channel Micromed EEG (Handy EEG SD32) and SystemPlus Evolution (Micromed SpA, Treviso, Italy) acquisition software. The recordings were obtained using a 22-bit sigma-delta A/D converter, with a sampling frequency (Fs) of 2048 Hz, an antialiasing band-pass filter set at 0.15–537.53 Hz, and a notch filter (50 Hz). Electrode impedances were kept below 10 kΩ to ensure that background noise in the acquired signal was <0.5 μV. The number of channels employed were, three for EEG, two for electrooculogram (EOG), and two for electromyogram (EMG). EEG (C3, C4, and Cz) was continuously recorded using an elastic cap, fitted with Ag/AgCl-positioned electrodes, as per the 10/20 system. FPz and Pz were the reference and ground electrodes, respectively. To monitor horizontal and vertical eye movement, the EOG signal was obtained via two electrodes, one placed above the outer canthus of the right eye and the other placed below the outer canthus of the left eye. The EMG signal was obtained via two surface electrodes (one active electrode and one reference electrode) located on each forearm, above the extensor carpi radialis longus muscle.

During all recordings, the laboratory lights were turned off and rechargeable batteries were used in the acquisition equipment to minimize potential AC induction at 50 Hz in the EEG power cables [47].

### 2.3. Data Analysis

**Figure 1:**
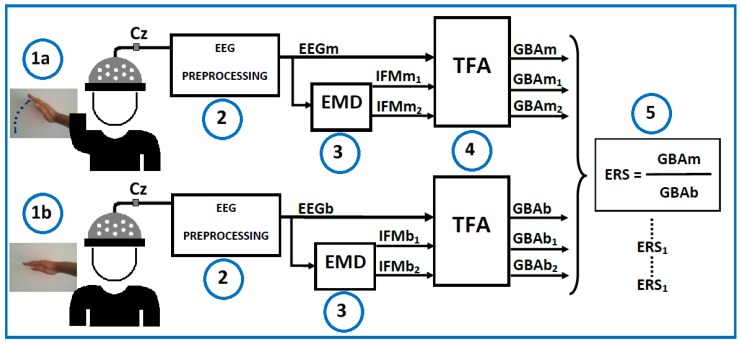
Simplified model of the event-related synchronization (ERS) calculation. Cz (electrode Cz from electroencephalogram (EEG) 10–20 system). (1a) Motor task; (1b) Basal activity; (2) Acquisition and analysis of the EEG signal (pre-processing); (3) Empirical Mode Decomposition (EMD) analysis of the EEG signal; (4) Time-frequency analysis (TFA) to obtain Gamma-band activity (GBA); (5) Calculation of the ERS.

The EEG, EMG, and EOG signals were analyzed, offline, using MATLAB 2016b (The MathWorks Inc., Natick, MA, USA) and FieldTrip [48], and the data were processed in European Data Format (EDF). The EEG signal measured on the Cz channel was analyzed. Figure 1 shows, block-by-block, the ERS computations.

The EEG pre-processing comprised a filter and artifact rejection stages. The signal was band-pass filtered (1–100 Hz) and narrow-band notch filtered (49–51 Hz eliminated band). Then, each trial was visually inspected using the FieldTrip ft_RejectVisual function to eliminate amplitude jumps, eye blinks, and muscle movements that were not previously eliminated using automatic analysis (FieldTrip ft_RejectArtifact). Next, the linear trend error (FieldTrip ft_Detrend) was eliminated. The output of the EEG pre-processing block was noted as *x*(*t*).

### 2.4. EMD Analysis of the EEG Signal

EMD decomposes a non-periodic and non-stationary signal into a finite number of intrinsic mode functions (IMFs). The IMFs must satisfy two conditions: (1) the number of extremes and the number of zero crossings are equal, or differ by no more than one, in the whole dataset; and (2) the mean value of the envelope defined by the local maximum and the envelope defined by the local minimum is zero at any point (IMFs are nearly periodic functions with zero mean). The original signal (*x*(*t*)) can be expressed as the sum of a finite number of IMFs and a residual

(1) $$x\left(t\right)=\overset{N}{\underset{j=1}{\sum }}{\mathrm{IMF}}_{j}+r_{N}\left(t\right),$$

where *N* denotes the total number of IMFs, IMF_j_ is the *j*th intrinsic mode function, and *r_N_* is the residue selecting N IMFs. EMD was applied to the *x*(*t*) signal following four steps: (i) Find all extreme points (maxima and minima) of *x*(*t*); (ii) generate the upper and lower envelopes (UE and LE, respectively) by interpolation of the maxima and minima with a cubic spline; (ii) compute the mean: M(*t*) = (UE + LE)/2; and (iv) subtract the mean from the original signal: *c*(*t*) = *x*(*t*) − *M*(*t*).

This process is iterated until the resulting signal *c*(t) complies with the criteria of an intrinsic mode function. Then, IMF1 = *c*(*t*) and the residue *r*_1_(*t*) = *x*(*t*) − *c*(*t*) is the new input signal for step (i), described above, (*x*(*t*) = *r*_1_(*t*)).

The number of extreme points decreases as the number of previous loop iterations increases. This algorithm stops when: (1) *r*(*t*) contains one extreme (maximum or minimum); (2) the computed IMF or the residual are too small; or (3) when five IMFs are computed.

The performance of Empirical Mode Decomposition is similar to that of a bank filter, where each IMF is bandwidth limited and can be identified as one of the frequency bands used in research and clinical practice. The only condition is that the sample frequency must be nearly five times that of the highest frequency of interest [49].

**Figure 2:**
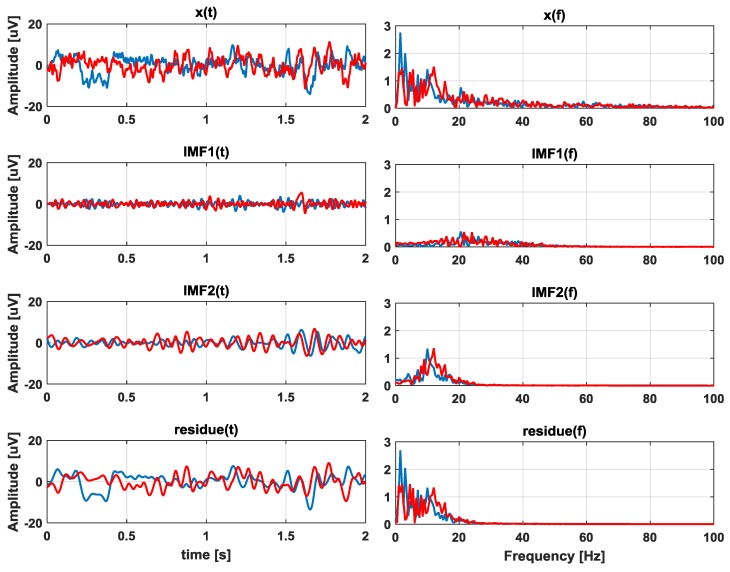
Example of electroencephalogram (EEG) signal decomposed into Intrinsic Mode Functions (IMF) IMF1, IMF2, and a residue. The left side is the time domain and the right side is the frequency domain. The red line depicts the basal activity, and the blue line is the motor activity (uV = micro Volts).

The EMD method estimates high-frequency modes first, since it fits envelopes through local minima and maxima. In Reference [50], the authors demonstrated that IMF1 represents the gamma band neuronal oscillation (>30 Hz), IMF2 represents beta band oscillation (13–30 Hz), IMF3 reflects the alpha band oscillation (8–13 Hz), IMF4 reflects the delta band oscillation (3.5–8 Hz) and IMFs 5 and 6 represent the theta band oscillation (0.5–3.5 Hz). Other works, such as References [51,52], also support the idea that IMF1 reflects the gamma band in EEG signals. Selecting the first IMF is comparable to a high-pass filtering based on Fourier methods. Unlike Fourier filtering, EMD does not remove components below an arbitrary cut-off frequency, so the gamma-band main contributions may vary between IMF1 and IMF2 [40]. An example of an EEG signal decomposed into IMF1 and IMF2 is shown in Figure 2 (with their corresponding frequency spectrums).

### 2.5. Calculation of TFA-GBA

The induced and spontaneous GBA were obtained from signals *x*(*t*), IMF1(*t*), and IMF2(*t*) as power spectral values and were estimated using a multi-taper fast Fourier transform (FFT) (FieldTrip ft_freqanalysis function). The time-frequency analysis (TFA) computed the mean PSD value (in µV^2^) in the frequency band (30–60 Hz). PSD reflected the amplitude of the neuronal oscillations, calculated using the time-frequency transformations [6]. GBA parameters were calculated for each individual trial and were averaged for each hand. An overall average was calculated for all subjects. Table 1 shows the parameters obtained in each experiment.

Once the basal GBA and motor GBA values were obtained for the right and left hands, ERS was defined by normalizing the values in relation to basal gamma activity and were presented as a percentage. This established the ERS as Equation (2) for the original signal *x*(*t*), as Equation (3) for the signal IMF1 (*t*), and as Equation (4) for the signal IMF2 (*t*).

(2) $$\mathrm{ERS}\left(\%\right)=\frac{\mathrm{GBAm}-\mathrm{GBAb}}{\mathrm{GBAb}}\times 100,$$

(3) $$ERS1\left(\%\right)=\frac{\mathrm{GBAm}1-\mathrm{GBAb}1}{\mathrm{GBAb}1}\times 100,$$

(4) $$ERS2\left(\%\right)=\frac{\mathrm{GBAm}2-\mathrm{GBAb}2}{\mathrm{GBAb}2}\times 100,$$

Negatives values of ERS were obtained when the basal activity was higher than the motor activity. These ERS parameters were calculated for each hand of each subject. The total average was calculated for all subjects.

### 2.6. Statistical Analyses

All statistical analyses were performed using the SPSS 22.0 program (SPSS Inc., Chicago, IL, USA). Data were tested using the Kolmogorov and Smirnov methods to determine whether they followed a Gaussian distribution. The data sampled from the Gaussian distribution were compared using the paired Student’s *t*-test, and non-Gaussian data were compared using the Wilcoxon test (non-parametric). Results are expressed as the mean and confidence interval (CI 95%). The significance value for the differences was set at *p* < 0.05.

## 3. Results

Table 2 shows the values (the total average for the 25 subjects) for the PSD of the basal GBA (GBAb, GBAb_1_, and GBA_2_) for the calculated frequency band.

Table 3 shows the values (the total average for the 25 subjects) for the PSD of the motor GBA (GBAm, GBAm1, and GBAm2) for both hands.

The ERS values obtained from the original pre-processing signal (*x*(*t*)) were compared with ERS1 and ERS2, obtained from the EMD method (IMF1 (*t*), IMF2 (*t*)). Table 4 shows these values for each subject of the study.

When the data were analyzed subject by subject, in 18 of the 25 cases, the best method was IMF1 for both hands. In six subjects (4, 7, 10, 19, 21, and 25), IMF2 was the best method for both hands, and for one subject (23) the best method was different for each hand.

Higher values of ERS were found in the right hand in right-handed subjects. This is because the more right-handed the subject is, the greater the area of the cerebral cortex used to move the right hand as compared with the left hand [29]. The same applies for ERS values in the left hand in left-handed subjects. For example, subject number 12 is left-handed and ERS1^RH^ * = 35.8% < ERS1^LH^ * = 68.4% and subject number 6 is right-handed and ERS1^RH^ * = 429.4% > ERS1^LH^ * = 294.1%.

**Figure 3:**
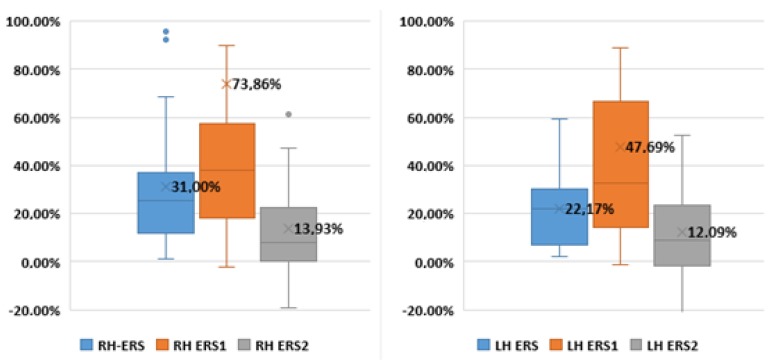
ERS values. RH = Right Hand, LH = Left Hand. To a better visualization, high outlier values have been removed.

Figure 3 and Table 5 present the average and the statistic ERS values. As a result of the pre-processing of EEG signals using IMFs, the GBA detection was improved for both the right and left hands: ERS1 > ERS (*p* < 0.05) and ERS1 > ERS2 (*p* < 0.05). ERS1 improves from 31.00% to 73.86% of the original ERS results for the right hand and from 22.17% to 47.69% of the original results of the left hand. The grand average value of ERS2 is significantly lower than that of ERS in both hands (ERS < ERS2, *p* < 0.05). As many of the subjects were right-handed (19 of 25), the highest average values were obtained for the right hand compared to the left hand for the typical method (ERS^RH^ = 31.0% > ERS^LH^ = 22.17%) for ERS1 (ERS1^RH^ = 73.86% > ERS1^LH^ = 47.69%) and for ERS2 (ERS2^RH^ = 13.93% > ERS2^LH^ = 12.09%).

## 4. Discussion

The purpose of this paper was to apply an EMD method to EEG signal processing and to evaluate whether the obtained IMFs improved the analysis of GBA using only one channel. This method is based on filtering the EEG signal using empirical model decomposition (EMD), after artifact rejection, and before obtaining the PSD values (Figure 1, step 3). The performance of this new method (band pass and notch filter + artifact rejection + EMD decomposition + TFA) was evaluated via comparison with the typical method (band pass and notch filter + artifact rejection + TFA).

The method with the highest GBA values (computed as motor/basal PSD ratio, ERS) was considered the best method for use in clinical purposes. ERS1 improves, from 31.00% to 73.86%, the results of the original ERS for the right hand, and from 22.17% to 47.69% of the original results for the left hand. Significantly higher values were obtained when IMF1 was used rather than those of the typical method for both the right (ERS = 31.00%, ERS1 = 73.86%, *p* < 0.05) and left hands (ERS = 22.17%, ERS1 = 47.69%, *p* < 0.05). However, significantly lower values were obtained when IMF2 was used, as opposed to those of the typical method, for right (ERS = 31.00%, ERS2 = 13.93%, *p* < 0.05) and left hands (ERS = 22.17%, ERS2 = 12.09%, *p* < 0.05). In all cases (subjects and hands), the EMD method (ERS1 or ERS2) values were higher than the ERS values.

These results demonstrated that using EMD in pre-processing enhanced GBA detection and ERS computation. The decomposition, based on direct extraction of the signal energy in IMFs, is particularly helpful for non-stationary signal evaluation compared to those used in GBA and ERS. Better computation of ERS allows researchers to achieve better quantification of changes in the activity of local interactions between main neurons and interneurons, which control the frequency components of the in-progress EEG. The use of EMD in the process of quantifying gamma-band activity could be transduced in an improvement of the diagnosis of functional deficits in patients with cerebrovascular disorders and Parkinson′s disease [30].

**Figure 4:**
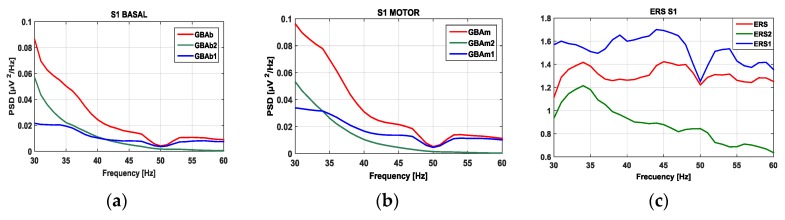
GBA signals from the right hand of subject S1: (**a**) Basal activity spectrum; (**b**) motor activity spectrum; (**c**) ERS spectrum.

The differences between ERS1 (obtained from IMF1) and ERS2 (obtained from IMF2) can be explained if EMD is interpreted as an adaptive filter [53]. The extracted IMFs are in decreasing order of frequency. The gamma-band can be considered “high EEG frequencies”, so it makes sense that the first components of the EMD decompositions are associated with GBA. The EMD decomposition is intrinsic to the frequency components of the signal registered, thus, in subjects with high frequency components (above 60 Hz), IMF1 will be above the range and IMF2 will suit the gamma band under consideration (30 to 60 Hz). As an example, Figure 4 shows the PSD waveforms and ERS waveform for subject 1 (S1) in the 30–60 Hz band. In this case, it is clear that GBAb2 is higher below the 30 Hz frequency limit. The relationship between IMF1 and GBA has been found in previous works [50,51,52].

The ERS values obtained using the original method agree with the results obtained in previous works: ERS ≈ 10–20% [54,55]. More recent studies [56] observed an increase in gamma-band activity in different tasks (emotional stimuli, face recognition, and motor control) using invasive techniques, such as electrocorticography. In our case, researchers obtained satisfactory results using a non-invasive technique.

From a neurophysiological point of view, it is important to remark that FPz has been selected as the reference because it is a midline electrode; thus, it is minimally contaminated by muscle artifacts [57]. One known problem of taking this point as reference is that the measured levels of GBA are low when Cz (active) minus FPz (reference) is computed. However, in this work, researchers compared the ratio between the motor activity and basal activity, so both parameters are equally affected by this fact, and the final ratio is unaltered.

EEG datasets obtained from setups with high numbers of EEG channels comprise a high dataset dimension, and this directly increases the computational complexity of performing further signal processing methods [58]; which further increases the time required to prepare the test. In this case, minimum complexity was been achieved using only one electrode.

## 5. Conclusions

As new methods to process and filter EEG signals are developed, the detection of gamma-band activity in motor tasks is improved. This study described how IMFs are effective in assessing GBA as a complement to Fourier-based methods. The ratio of motor and basal activity obtained using IMFs improved, from 31.00% to 73.86% of the original value, for the right hand, and 22.17% to 47.69% for the left. The new method can improve the study of cerebral motor areas and the assessment of neural diseases (vascular, traumatic, and degenerative pathologies). Complexity and testing time in clinical practice can be reduced because only one EEG channel is used.

## Figures and Tables

**Table 1:** Experimental parameters.

Experiment	Gamma Oscillations	Waveform *	TFA Output µV^2^	Processing Method
Basal (b) activity	Spontaneous	*x*(*t*) = EEGb	GBAb	Typical
		IMFb1	GBAb_1_	Based on IMF1
		IMFb1	GBAb_2_	Based on IMF2
Motor (m) activity	Induced	*x*(*t*) = EEGm	GBAm	Typical
		IMFm1	GBAm_1_	Based on IMF1
		IMFm2	GBAm_2_	Based on IMF2

* As defined in Figure 1.

**Table 2:** Average basal Gamma-band activity (GBA) values (Power Spectrum Density (PSD) in μV^2^), expressed as the mean (CI 95%).

GBAb	GBAb_1_	GBAb_2_
0.0151	0.0059	0.0072
(0.0121–0.0180)	(0.0045–0.0072)	(0.0058–0.0086)

**Table 3:** Average motor GBA values (PSD in μV^2^), expressed as the mean (CI 95%).

Case	Left Hand	Right Hand
GBAm	0.0186	0.0194
	(0.0146–0.0225)	(0.0156–0.0232)
GBAm1	0.0081	0.0088
	(0.0064–0.0098)	(0.0071–0.0105)
GBAm2	0.0082	0.0083
	(0.0063–0.0100)	(0.0063–0.0104)

**Table 4:** Study subjects detailed for each case. Bold results are the highest results for each subject.

		Right Hand			Left Hand		
		Typical	Based on IMFs		Typical	Based on IMFs	
Subject	Laterality *	ERS^RH^ *	ERS1^RH^ *	ERS2^RH^ *	ERS^LH^ *	ERS1^LH^ *	ERS2^LH^ *
1	A	30.7%	54.6%	3.5%	57.5%	67.6%	43.0%
2	R	32.0%	48.6%	22.6%	3.9%	5.4%	3.8%
3	R	13.0%	16.7%	6.7%	7.2%	13.3%	−3.3%
4	R	35.6%	15.6%	61.3%	17.8%	9.4%	33.3%
5	R	95.4%	291.7%	1.7%	23.1%	88.9%	3.4%
6	R	68.5%	429.4%	−19.3%	41.3%	294.1%	−23.8%
7	R	1.0%	−2.0%	5.4%	2.0%	0.0%	2.7%
8	R	25.3%	40.8%	17.0%	24.7%	46.0%	10.2%
9	R	9.4%	37.9%	−16.4%	17.2%	44.8%	−7.3%
10	L	1.7%	−2.0%	7.4%	23.3%	24.0%	25.9%
11	R	23.0%	47.4%	1.1%	18.4%	32.2%	7.0%
12	L	30.3%	35.8%	22.3%	59.1%	68.4%	46.2%
13	R	20.2%	25.8%	13.0%	22.2%	23.2%	19.3%
14	R	38.0%	59.7%	24.2%	34.1%	56.8%	19.7%
15	R	11.0%	29.1%	−1.0%	14.3%	35.6%	3.8%
16	R	27.8%	41.1%	15.8%	26.4%	32.6%	20.4%
17	L	24.6%	35.7%	13.0%	6.4%	14.9%	−0.5%
18	R	92.3%	296.8%	−10.4%	19.2%	65.0%	−4.3%
19	R	60.3%	26.0%	104.0%	38.2%	28.6%	52.7%
20	R	1.4%	51.8%	−19.3%	5.0%	49.5%	−14.3%
21	R	1.6%	−0.8%	7.8%	2.5%	−1.4%	9.0%
22	A	13.4%	89.6%	−10.8%	5.8%	76.0%	−15.9%
23	R	32.9%	19.6%	47.2%	22.7%	26.4%	21.2%
24	R	64.7%	146.5%	18.1%	37.1%	76.6%	13.4%
25	A	21.1%	11.1%	33.4%	24.4%	14.0%	36.5%

* RH = Right Hand, LH = Left Hand. Laterality: A = Ambidextrous, R = Right-handed, L = Left-handed.

**Table 5:** ERS values.

Hand		N	TEST	*p*
Right hand	ERS and ERS1	25	T-Student	0.023
	ERS and ERS2	25	T-Student	0.021
	ERS1 and ERS2	25	T-Student	0.020
Left hand	ERS and ERS1	25	T-Student	0.001
	ERS and ERS2 *	25	Wilcoxon	0.006
	ERS1 and ERS2 *	25	Wilcoxon	0.002

* ERS2 data follows a non-Gaussian distribution.
